# Talocalcaneal Coalition: Current Concepts, Clinical Implications, and Management Strategies

**DOI:** 10.3390/life16030495

**Published:** 2026-03-18

**Authors:** Antonio Mascio, Chiara Comisi, Virginia Cinelli, Federico Moretti, Gloria Assegbede, Giulio Maccauro, Tommaso Greco, Carlo Perisano

**Affiliations:** 1 Department of Orthopedics and Geriatric Sciences, Catholic University of the Sacred Heart, Largo Francesco Vito 8, 00168 Rome, Italy; antonio.mascio87@gmail.com (A.M.);; 2 Department of Orthopedics, Ageing and Rheumatological Sciences, Fondazione Policlinico Universitario A. Gemelli IRCCS, Largo Agostino Gemelli 8, 00168 Rome, Italy; 3 Department of Life Sciences, Health, and Healthcare Professions, Link Campus University, 00165 Rome, Italy

**Keywords:** talocalcaneal coalition, synostosis, synchondrosis

## Abstract

Talocalcaneal coalition is a frequent cause of painful rigid flatfoot in adolescents and young adults, resulting from congenital failure of segmentation with fibrous, cartilaginous, or osseous bridging of the subtalar joint. Clinical presentation typically coincides with skeletal maturation and includes hindfoot pain, recurrent ankle sprains, progressive stiffness, and characteristic planovalgus deformity. Although prevalence is likely underestimated, advances in imaging have improved recognition and characterization. Diagnosis relies on the integration of clinical findings with imaging, where computed tomography (CT) remains the reference standard, while magnetic resonance imaging (MRI) enables accurate detection of both osseous and non-osseous coalitions and associated soft-tissue changes. This narrative review aims to provide a comprehensive and updated synthesis of current concepts in talocalcaneal coalition, with specific focus on its clinical implications and contemporary management strategies. We critically analyze diagnostic pathways, including emerging modalities such as weight-bearing CT, and discuss evidence-based indications for conservative treatment, coalition resection, and arthrodesis. Particular attention is devoted to patient selection, prognostic factors, and evolving minimally invasive techniques. Current limitations and areas of controversy are highlighted, emphasizing the need for standardized imaging criteria and optimized treatment algorithms to improve long-term functional outcomes.

## 1. Introduction

The Talocalcaneal (TC) coalition represents the second most frequent form of tarsal coalition, ranking immediately after the calcaneonavicular (CN) variant in terms of occurrence and clinical relevance [1]. This pathological condition consists of an abnormal structural connection between the talus and the calcaneus, which progressively alters the integrity and function of the medial longitudinal arch. As the deformity evolves, it leads to the development of pes planus and a gradual restriction of subtalar joint motion, ultimately impairing foot mechanics and gait. Over time, these biomechanical changes contribute to pain, stiffness, and difficulty in performing high-impact activities such as running or jumping.

TC coalition produces a significant alteration in hindfoot biomechanics by restricting motion at the subtalar joint. The abnormal union between the talus and calcaneus limits or completely blocks subtalar mobility, resulting in reduced inversion and eversion of the hindfoot and a consequent increase in hindfoot rigidity. Under physiological conditions, the subtalar joint plays a key role during gait: during the loading response phase, hindfoot eversion allows the foot to adapt to the ground and contributes to shock absorption, whereas during the pre-swing phase inversion converts the foot into a more rigid lever for propulsion. In the presence of talocalcaneal coalition, this mechanism is impaired. The absence or reduction in early eversion limits the foot’s ability to accommodate uneven surfaces and reduces shock attenuation, leading to increased transmission of forces to adjacent structures such as the ankle, midfoot, and knee. As a consequence, patients may experience pain during walking and an altered gait pattern. Furthermore, the rigidity of the subtalar joint leads to compensatory biomechanical adaptations, including increased stress at the tibiotalar joint and greater motion at the midfoot, with subsequent alterations in plantar loading distribution. Over time, these changes may contribute to the development of rigid flatfoot deformity, recurrent ankle sprains, and secondary degenerative changes.

This coalition is a well-recognized etiology of painful flatfoot in older children and adolescents, resulting specifically from an anomalous fusion that may manifest in three distinct histological forms—fibrous (syndesmosis), cartilaginous (synchondrosis), or osseous (synostosis)—depending on the degree of tissue differentiation and maturation [2]. Although the underlying defect is congenital, clinical symptoms rarely appear during early childhood. Instead, they generally emerge only when the initially flexible fibrous bridge progressively transforms into a stiffer cartilaginous bar and eventually ossifies, forming a rigid bony coalition. This transition reduces joint elasticity and produces the characteristic mechanical block of the subtalar joint. Symptom onset most commonly occurs between the ages of 12 and 15 years, a period corresponding to the phase of skeletal maturation and increased physical activity [1,3].

The anatomical and clinical aspects of TC coalition were first systematically described by Zuckerkandl in 1877, who provided an early morphological account of the condition. In the following decades, additional contributions by Slomann (1921) [4], Badgeley (1927) [5], and subsequently Harris and Beath (1948) [6] expanded the understanding of this entity, emphasizing its clinical significance. Their pioneering works established the link between tarsal coalitions and the development of the peroneal spastic flatfoot, thereby framing TC coalition as a distinct pathological condition rather than a simple anatomical curiosity [7,8].

From an anatomical perspective, the subtalar joint is composed of three articular facets—anterior, middle, and posterior—that together facilitate complex triplanar motion, essential for foot adaptability during gait. The middle facet has traditionally been considered the most frequently involved site of TC coalition, although more recent imaging and cadaveric studies have suggested that the posterior facet may actually be affected with comparable or even greater frequency [9,10]. The specific morphology and localization of the coalition play a crucial role in determining the resulting foot architecture: involvement of the middle facet is most commonly associated with the collapse of the medial arch and the development of pes planus, whereas posterior facet coalitions can occasionally manifest with an opposite pattern, leading to a mild cavus or neutral foot alignment [11]. The previously reported prevalence of TC coalition, estimated between 1% and 2%, is now widely regarded as a significant underestimation due to limitations in early diagnostic modalities. With the advent of advanced cross-sectional imaging, particularly computed tomography (CT) and magnetic resonance imaging (MRI), more recent investigations have revealed a much higher incidence. Nalaboff and Schweitzer, in a prospective MRI study involving 574 patients older than 12 years who underwent foot and ankle evaluation, identified a prevalence rate of 11.5%, thereby demonstrating the substantial underdiagnosis of this condition in clinical practice [12]. Similarly, an anatomical investigation examining 114 feet from 62 cadavers without any known history of foot pathology reported an even higher prevalence of 13%, reinforcing the concept that many coalitions remain clinically silent or are misinterpreted as other causes of foot pain or deformity [13]. Furthermore, approximately half of all documented cases are bilateral, which underscores the importance of assessing both feet when a coalition is suspected [14,15]. In 2008, Martus et al. [16] reported for the first time the association between an accessory anterolateral talar facet and symptomatic rigid flatfoot, identifying this anatomical variant as a novel etiology of painful TC impingement.

Given its underestimated prevalence, variable clinical presentation, and significant impact on foot biomechanics, TC coalition continues to represent a condition of considerable clinical importance. A comprehensive and multidisciplinary evaluation—integrating clinical examination, advanced imaging, and biomechanical assessment—is essential to ensure accurate diagnosis, appropriate classification, and individualized management. Improved awareness of its anatomical variability and radiologic spectrum is critical for optimizing treatment strategies, preventing long-term disability, and preserving functional outcomes in affected adolescents and young adults.

A narrative literature review on talocalcaneal coalition was conducted using the main medical databases, including PubMed, Scopus, and Web of Science. The search included articles published in English up to 2024 using the keywords “talocalcaneal coalition”, “tarsal coalition”, “subtalar coalition”, “resection”, and “arthrodesis”. Studies addressing epidemiology, diagnosis, imaging findings, and treatment strategies were considered. Relevant publications were selected based on their scientific relevance and were qualitatively analyzed to summarize current evidence.

## 2. Diagnosis

The diagnosis of TC coalition requires the integration of a thorough clinical evaluation with appropriate imaging studies, as neither alone is sufficient in all cases. From a clinical perspective, patients may present with a spectrum of symptoms, most commonly medial or retro-malleolar pain that worsens with activity, recurrent ankle sprains related to subtalar instability, and progressive restriction of inversion–eversion movements at the subtalar joint. Over time, these alterations can contribute to the development of a rigid flatfoot, which is often the clinical hallmark prompting further investigation. Nevertheless, it is important to recognize that a considerable proportion of individuals with talocalcaneal coalition remain entirely asymptomatic or display only subtle and nonspecific signs, which may delay diagnosis or lead to incidental detection [17,18]. Historically, peroneal spasm was regarded as a pathognomonic feature of this condition; however, more recent studies have clarified that true peroneal spasm is in fact uncommon and should not be considered a reliable diagnostic marker [19].

### 2.1. Conventional Radiographs

**Figure 1:**
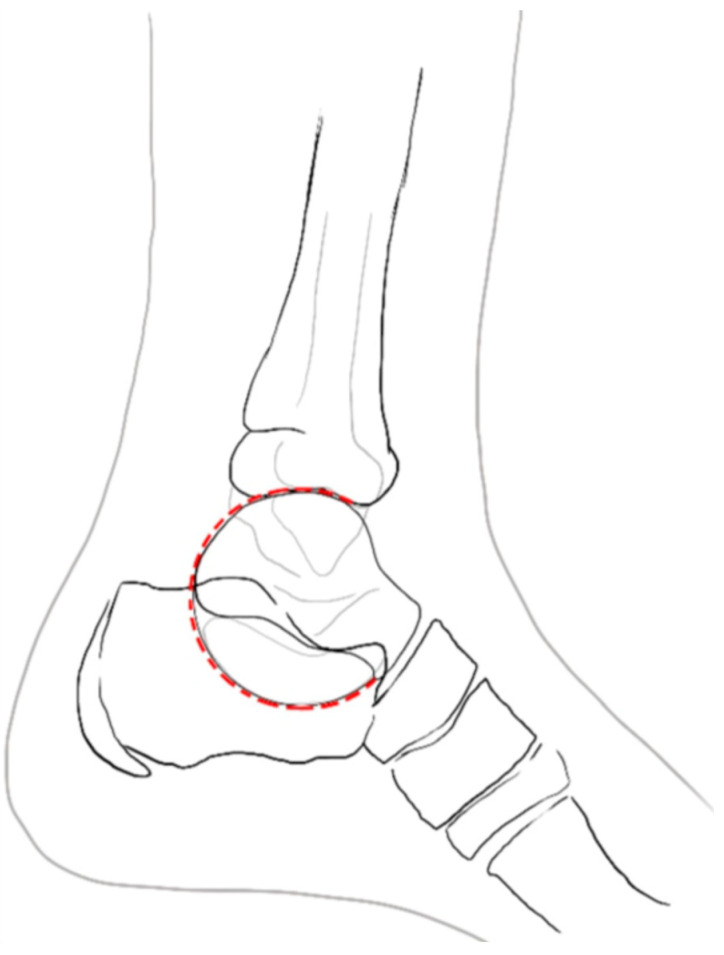
C-sign.

Weight-bearing radiographs remain the initial diagnostic step. Standard antero-posterior (AP), lateral, and oblique weight-bearing views represent a fundamental first-line diagnostic tool and are often indispensable in confirming coalition or other foot pathologies [20,21]. Sensitivity of unenhanced radiographs for TC coalitions reached 100% with 88% specificity, while CN coalitions showed 80–100% sensitivity and 97–98% specificity. Newly described signs, including sustentaculum-tali dysmorphia, non-visualization of the middle facet, and talar neck shortening, significantly improved diagnostic accuracy, confirming plain radiographs as a reliable screening tool [22]. On the lateral projection, the “C-sign”, a continuous arc formed by the medial talar outline and the inferior cortex of the sustentaculum-tali, is a well-described marker of TC coalition [23] (Figure 1).

However, its sensitivity and specificity vary substantially, depending on patient age and coalition site, and false positives may occur in normal feet [24]. Other indirect signs include talar beaking, non-visualization of the middle facet, and sustentaculum tali dysmorphia (often described as “brick-like” morphology) [25].

Furthermore, in pediatric cohort the C-sign alone showed a sensitivity of only ~54% and specificity of 86%, whereas ultrasonography achieved a sensitivity of 96% and specificity of 98% compared to 3D CT as the reference standard [26].

**Figure 2:**
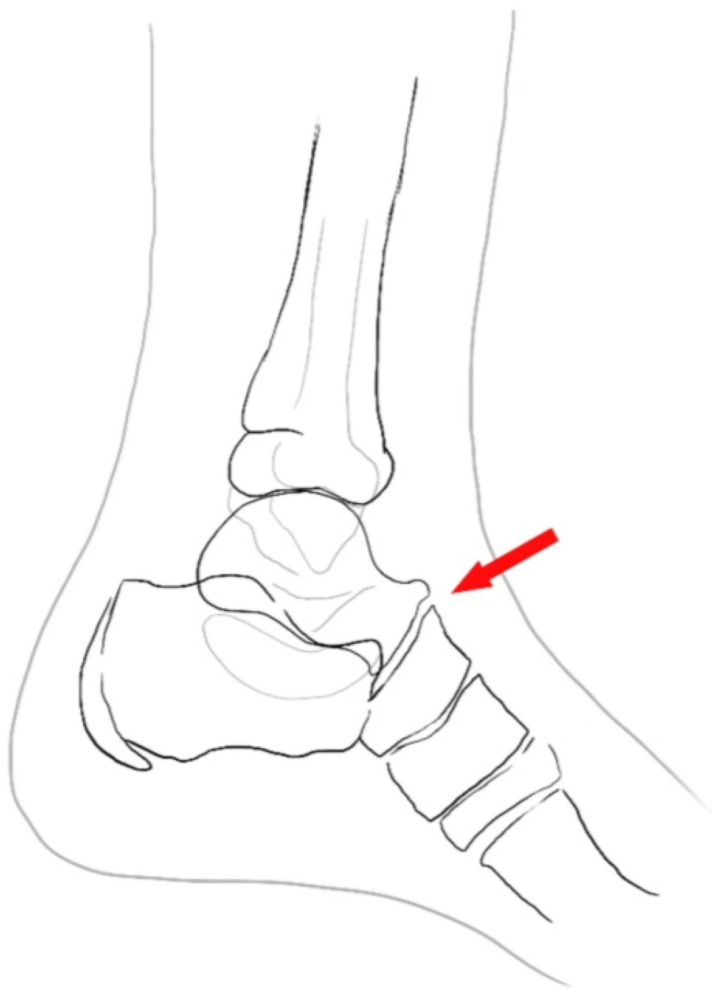
Talar beak-sign.

Another finding in the lateral projection is a “talar beak sign”, defined as a superior flaring of the talar head on lateral views, which develops secondary to restricted subtalar motion and navicular overriding of the talus; it is often associated with limitation of dorsiflexion and significant pain [27] (Figure 2).

In addition to the classical radiological findings typically associated with TC coalition, two less commonly discussed but highly characteristic imaging signs have been described in the literature: the “humpback sign” and the “duck-face sign” [28]. The humpback sign consists of an osseous protuberance on the posterior aspect of the subtalar joint, visible on lateral radiographs or sagittal CT images. It results from chronic mechanical stress and limited subtalar motion due to the coalition, creating a characteristic “hump” appearance. This indirect sign suggests subtalar rigidity and should prompt further imaging with CT or MRI to confirm the presence and extent of the coalition.

The duck-face sign refers to a bony prominence on the medial TC joint, projecting over the lower half of the medial malleolus. On oblique or coronal images, it resembles a “duck beak,” reflecting hypertrophic changes from abnormal loading at the coalition site. It is a useful indicator of medial facet involvement, especially when the coalition is non-ossified and poorly seen on plain radiographs.

### 2.2. Computed Tomography

When suspicion remains, CT is regarded as the gold standard for defining morphology, extension, and facet involvement [29,30]. Its multiplanar reconstructions and 3D views allow classification based on type (overgrowth of talus/calcaneus, accessory ossicles, complete coalition) and on the facet involved (anterior, middle, or posterior), even if in coalitions of the middle and posterior facets, the sinus tarsi is not visible on 3D reconstruction [31,32]. CT is also indispensable for preoperative planning, providing accurate assessment of coalition size and osseous anatomy. In 1992, Kumar et al. [33] identified three types of TC coalition on preoperative CT that correlated with operative findings: Type I, osseous bridging of the middle facet; Type II, cartilaginous narrowing with cortical irregularity; and Type III, fibrous narrowing of the middle facet. Importantly, the type of coalition did not appear to influence surgical outcomes. CT scanning offers precise assessment of the osseous or cartilaginous nature of talocalcaneal coalitions and facet orientation, improving both diagnosis and preoperative planning. Two-and three-dimensional reconstructions allowed identification of new peripheral posterior bony coalitions (Type V), coalitions aligned with standard CT or Harris view cuts (Type I), and complex vertical or combined horizontal–vertical fibrocartilaginous types (Type I and II). CT also enables accurate sizing of complete bony coalitions (Type IV), which is crucial in determining resectability [32].

### 2.3. Magnetic Resonance Imaging

A large MRI-based study revealed that tarsal coalitions are significantly more common than previously reported, with an overall prevalence of about 11% among patients undergoing ankle MRI [12].

MRI has emerged as a highly accurate modality for identifying both osseous and non-osseous coalitions. In a recent diagnostic accuracy study including 168 MRIs (56 confirmed talocalcaneal coalitions, 112 controls), MRI achieved a pooled sensitivity of 95.8% and specificity of 94.3%, with near-perfect interobserver agreement (κ = 0.895) [34]. In addition to characterizing fibrocartilaginous components, MRI can demonstrate bone marrow edema, cartilage changes, joint effusion, and concomitant osteochondral lesions, particularly valuable in children where CT may underestimate cartilaginous involvement [32]. A recent study established reproducible MRI-based guidelines to measure the size of talocalcaneal coalitions, demonstrating good to excellent inter-rater reliability. These standardized coronal Proton Density PD MRI measurements provide a reliable method to quantify coalition size for diagnostic and preoperative planning [35]. Accurate assessment of site and extent of TC coalition is essential for treatment planning. In a small series, both CT and MRI effectively characterized coalition type and extension, but MRI proved superior by detecting associated subchondral changes not visible on CT, highlighting its added value over conventional imaging [36] (Table 1).

### 2.4. Emerging and Alternative Modalities

**Figure 3:**
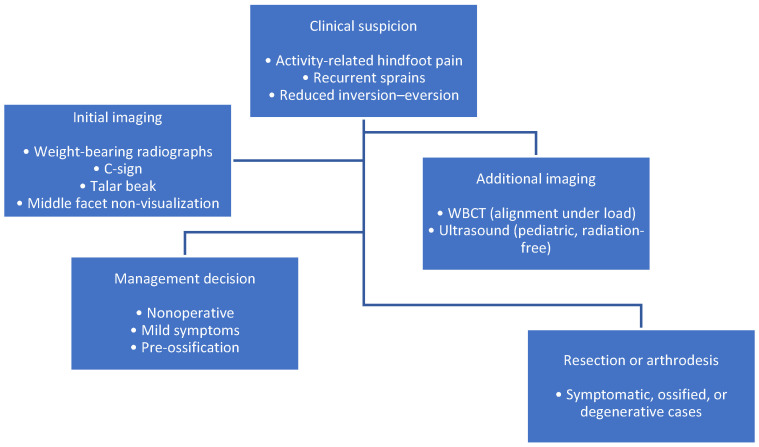
Summary of current management strategies for talocalcaneal coalition. A schematic summary illustrating the stepwise diagnostic approach. Clinical suspicion (activity-related hindfoot pain, recurrent sprains, limited inversion–eversion) leads to initial imaging with weight-bearing radiographs (C-sign, talar beak). Cross-sectional imaging with CT and MRI defines morphology and tissue composition, while adjuncts such as weight-bearing CT and ultrasound assist in selected cases. The resulting diagnostic synthesis guides management decisions toward conservative or surgical treatment.

Weight-bearing CT has been recently introduced to evaluate hindfoot alignment under physiological load and to quantify the biomechanical impact of coalitions [34]. Ultrasound has also shown promising results in pediatric populations, offering radiation-free visualization of non-ossified coalitions, although operator dependency remains a limitation [26] (Figure 3).

## 3. Treatment

The management of TC coalition has evolved significantly over the last century, reflecting a better understanding of the natural history of the condition and the biomechanical consequences of restricted subtalar motion (Table 2). Early reports by Harris and Beath in 1948 were among the first to emphasize the role of coalitions in rigid flatfoot and peroneal spasm, recommending surgical resection in symptomatic patients as a way to restore subtalar mobility and alleviate pain [6]. Badgeley and Slomann had previously noted the importance of these anomalies in rigid pes planovalgus deformities, laying the groundwork for surgical exploration [5,37].

### 3.1. Conservative Management

Nonoperative treatment remains the initial approach, particularly in children and adolescents with mild symptoms. This includes activity modification, analgesics or non-steroidal anti-inflammatory drugs (NSAIDs), physiotherapy focused on flexibility and strengthening, custom orthoses to support the medial longitudinal arch, and temporary immobilization in a cast or boot [7,38,39]. These strategies may reduce pain and improve function temporarily, but their long-term efficacy is limited, especially once the coalition ossifies.

### 3.2. Surgical Management

When conservative measures fail, surgery is the mainstay of treatment. Two principal surgical strategies are described: resection and arthrodesis.

• **Coalition resection**: First popularized in the early 20th century and later refined by Badgley and Harris, resection aims to remove the abnormal bridge and restore subtalar mobility [5,6]. Fat, muscle, tendon, or bone wax is commonly used for interposition to reduce recurrence [32,41]. According to Wilde [40], favourable outcomes after resection of TC coalition are expected when the coalition involves no more than 50% of the posterior facet of the calcaneus on coronal CT, heel valgus is less than 16 degrees, and there are no radiographic signs of subtalar arthritis. The presence of talar beaking, although frequently observed, does not appear to compromise the clinical result. Conversely, resection is associated with poorer outcomes when the coalition occupies more than 50% of the posterior facet, when heel valgus exceeds 16 degrees, and when degenerative changes or impingement of the lateral talar process on the calcaneus are present. Mubarak and Patel (2009) highlighted that resection is most effective in patients younger than 16 years, with coalitions involving <50% of the subtalar joint and without degenerative changes [49]. Long-term follow-up studies have reported return to sports in the majority of adolescent patients, especially when the middle facet is isolated [51]. However, recurrence, incomplete pain relief, and progressive deformity remain possible, particularly in large or posterior facet coalitions. In a series of 20 non-osseous TC coalitions assessed with 3D-CT, the posterior facet and total joint surface areas were significantly larger than controls (40% and 12%, respectively), and postoperative American Orthopaedic Foot and Ankle Society (AOFAS) scores improved in all resected cases, supporting resection as a valid option for non-osseous coalitions [47]. In the largest systematic review to date on adult TC coalition (72 patients), resection proved a safe option, with superior outcomes reported for endoscopic techniques or flexor hallucis longus tendon interposition, while conservative management showed limited benefit [50]. In a prospective series of 97 tarsal coalition resections (49 TC), patients achieved high satisfaction and functional improvement, with a mean return to activity at 18.3  ±  9.6 weeks and a Roles and Maudsley score of 1.3 at final follow-up [54].
• **Arthrodesis**: Subtalar or triple arthrodesis is reserved for severe, extensive, or degenerative coalitions, most often in older adolescents or adults [25]. This procedure reliably relieves pain and stabilizes the hindfoot, but at the expense of subtalar motion. Harris and Beath already noted in 1948 that arthrodesis provided durable symptom relief when resection was unlikely to succeed [6]. Triple arthrodesis is recommended in cases with >50% joint involvement, arthrosis, or marked hindfoot malalignment. In adolescents, the choice between resection and arthrodesis remains debated, but in adults with symptomatic coalitions unresponsive to conservative care, arthrodesis is generally required to achieve reliable pain relief and functional improvement [55]. According to Recordon et al. in pediatric and adolescent patients, most foot deformities can be managed conservatively or with joint-preserving procedures, but in severe fixed deformities triple arthrodesis remains a valuable option to relieve pain, correct alignment, and maintain a plantigrade foot [45].

### 3.3. Emerging Techniques

Endoscopic or arthroscopically assisted resections have recently been described, offering the potential advantages of smaller incisions, less soft-tissue disruption, and faster recovery [46]. Arthroscopic resection of TC coalition demonstrated favourable midterm results, with 81% of patients achieving good or excellent outcomes and significant improvements in Visual Analogue Scale (VAS) and AOFAS scores, alongside a low complication rate [57]. Percutaneous subtalar arthrodesis (PASTA) proved to be a safe and effective option for adult patients with symptomatic medial facet talocalcaneal coalition and normal hindfoot alignment, yielding significant clinical improvement and 100% fusion rates at 2-year follow-up [58].

A retrospective study of 16 patients with TC coalition treated between 2019 and 2023 demonstrated that total arthroscopic resection is a safe and effective alternative to open surgery. Significant improvements were observed in pain (VAS from 4.3 to 1.8) and function (AOFAS from 65.6 to 87.3), with high patient satisfaction and no recurrences on follow-up imaging. Only one case of postoperative numbness occurred, and mini-incisions were required in complex cases with tibial nerve compression [59].

### 3.4. Hindfoot Alignment and Adjunctive Procedures

An important aspect in the management of talocalcaneal coalition is the assessment and correction of hindfoot alignment, as this condition is frequently associated with planovalgus deformity and secondary biomechanical alterations of the foot. In symptomatic patients presenting with significant hindfoot valgus, medial arch collapse, or gastrocnemius–Achilles tightness, coalition resection alone may be insufficient to restore normal biomechanics and prevent persistent symptoms [40,56]. For this reason, several authors recommend addressing the underlying deformity through adjunctive procedures aimed at improving alignment and load distribution [54,60]. These may include calcaneal osteotomies to correct hindfoot valgus, Cotton osteotomy to restore medial column alignment, subtalar arthroereisis in selected pediatric patients, and gastrocnemius recession or Achilles tendon lengthening when equinus contracture is present [44,45,60,61]. The choice of additional procedures should be individualized based on the severity of deformity, patient age, skeletal maturity, and functional demands. Incorporating alignment correction into the surgical strategy may improve clinical outcomes, reduce the risk of persistent flatfoot deformity, and optimize long-term function following coalition resection [54,56,60].

### 3.5. Post-Operative and Complications

Post-operative outcomes following surgical treatment of talocalcaneal coalition are generally favourable, particularly when appropriate patient selection and meticulous surgical technique are applied; however, a spectrum of complications has been described in the literature. According to Mousa et al., who compared arthroereisis with corrective osteotomy combined with coalition resection, no statistically significant differences were observed between the two approaches in terms of functional outcomes, radiological results, or foot alignment, despite a higher complication rate in the arthroereisis group reaching up to 25% [42].

In a retrospective study involving 10 children (11 feet), early postoperative complications were limited to one case of superficial wound infection and one case of wound dehiscence, both successfully managed with oral antibiotic therapy. Late complications consisted of mild residual forefoot supination observed in two patients. No additional surgical procedures were required during the follow-up period [43]. In other retrospective study comparing operative and nonoperative treatment in 47 feet affected by talocalcaneal coalition, no complications were reported in either group; however, six patients (seven feet) managed conservatively required delayed surgical intervention due to persistent dissatisfaction, while percutaneous Achilles tendon lengthening was performed intraoperatively in four patients (five feet) to achieve at least 5° of ankle dorsiflexion [62]. Subtalar arthroereisis is a minimally invasive option for pediatric heel valgus with rapid recovery, but it may be associated with implant-related complications such as loosening, breakage, sinus tarsi pain, peroneal spasm, and joint effusion. Compared to corrective osteotomies, it shows lower surgical morbidity and shorter immobilization but carries a higher risk of device intolerance. Routine screw removal after two years may reduce implant failure and residual pain without significant recurrence of deformity [44,61]. Arthroscopic resection offers the advantage of smaller incisions and a lower complication rate compared to open surgery [51].

Talocalcaneal coalition may cause characteristic complications due to bony hypertrophy and articular protrusion, leading to compression of surrounding soft tissues. The most frequent complications observed were tibial nerve compression, synovial cyst formation, and displacement of adjacent tendons (FHL, FDL, and posterior tibial tendon). High-frequency ultrasonography proved highly effective in identifying these complications, particularly tibial nerve compression, while CT detected only a limited number of synovial cysts and conventional radiographs failed to demonstrate any associated soft-tissue complications, radiograph failed to show any complications of the TCC [52].

## 4. Discussion

The management of TC coalition remains complex, especially regarding the balance between resection and arthrodesis, and the debated role of interposition materials. This review provides an integrated and updated synthesis combining traditional clinical criteria with emerging imaging modalities and minimally invasive surgical approaches, offering a practical algorithm for patient-specific decision-making. Conservative measures are often attempted first, but many symptomatic patients ultimately require surgery, particularly in skeletally immature individuals or those with persistent pain and functional limitations [2,11].

Coalition resection has shown favourable outcomes when strict selection criteria are applied, as described by Wilde [40] and Comfort [56], who emphasized the importance of coalition size, hindfoot alignment, and absence of degenerative changes. Patients with coalitions involving less than 50% of the posterior facet and valgus <16° are more likely to achieve excellent or good outcomes, whereas larger coalitions with malalignment or arthrosis often progress to poor results, necessitating arthrodesis [40,56].

The role of interposition following resection remains uncertain. Historically, several materials have been used, including fat grafts, tendon or muscle flaps (e.g., extensor digitorum brevis), bone wax, and synthetic substitutes [48,53,60,63,64]. The recent systematic review by Spaans et al. [65] analyzed 21 studies with 436 patients (581 feet), including 153 TC coalitions, and found an overall recurrence rate of 9%, with 4% in TC and 11% in CN resections. Among interpositions, fat grafts showed a recurrence of about 7%, while tendon or muscle interposition had higher recurrence rates (up to 17% in CN cases). Bone wax produced very low recurrence rates but is associated with inflammatory risks [48]. Nevertheless, due to the low methodological quality of the included studies (median MINORS score 7), no definitive superiority of one material could be established [65].

For adolescents and adults, the decision between resection and arthrodesis remains controversial. While resection can preserve subtalar motion and allow return to sports [60,66], arthrodesis provides more predictable outcomes in cases with advanced degenerative changes or when more than 50% of the joint is involved [67,68]. Recent minimally invasive techniques, such as arthroscopic resection [63] and percutaneous arthrodesis [69], have shown promising mid-term results, but require further validation with larger cohorts and longer follow-up.

Overall, the literature suggests that resection remains the first-line surgical option in carefully selected young patients without degenerative disease, while arthrodesis should be reserved for older patients or those with extensive and rigid coalitions. Interposition may reduce recurrence, but current evidence does not support the superiority of one material over another. High-quality prospective studies are still needed to clarify optimal surgical strategies and the true role of interposition techniques [70,71,72].

## 5. Conclusions

TC coalition represents a clinically significant cause of pain, stiffness, recurrent sprain, limited range of motion of the foot, and deformities such as pes planovalgus in children, adolescents, and younger adults. Advances in diagnostic imaging have improved recognition of both osseous and non-osseous forms, yet treatment selection remains challenging. Resection provides excellent outcomes when strict criteria are met, particularly in skeletally immature patients with limited coalition size and without degenerative changes, while arthrodesis remains the preferred option in adults and in extensive or arthritic cases. The role of interposition following resection is still debated: although recurrence rates appear low overall, current evidence does not demonstrate clear superiority of any single material. Emerging minimally invasive approaches, including arthroscopic resection and percutaneous arthrodesis, show promising results but require further validation. Future high-quality prospective studies are needed to refine surgical indications, optimize patient selection, and establish evidence-based guidelines to improve long-term functional outcomes.

## Figures and Tables

**Table 1:** CT vs. MRI in the evaluation of talocalcaneal coalition.

Imaging Modality	Main Advantages	Indications	Key Findings	Limitations	Clinical Role
**CT**	High spatial resolution; excellent bony detail; 3D reconstructions	Preoperative planning; assessment of coalition size and morphology	Osseous bridging, facet involvement, coalition extent	Limited detection of non-osseous coalitions; radiation exposure	Precise anatomical definition and preoperative planning
**MRI**	Superior soft-tissue contrast; no radiation	Suspected fibrous/cartilaginous coalition; inconclusive radiographs/CT; pediatric patients	Non-osseous coalition, bone marrow edema, cartilage damage, joint effusion, osteochondral lesions	Less precise bony detail than CT; less optimal for surgical planning	Suspected non-osseous coalition or persistent symptoms despite negative/inconclusive radiographs or CT

**Table 2:** Diagnostic workflow Summary of current management strategies for talocalcaneal coalition, including indications, main surgical or conservative techniques, clinical outcomes, and limitations. Acronyms: AOFAS = American Orthopaedic Foot & Ankle Society score; VAS = Visual Analogue Scale for pain; FHL = Flexor Hallucis Longus; PASTA = Percutaneous Arthroscopic Subtalar Arthrodesis; NSAIDs = Non-Steroidal Anti-Inflammatory Drugs.

Approach	Indications	Main Techniques	Outcomes	Limitations
**Conservative management**	Children/adolescents with mild symptoms; early stages before ossification [5,7,38]	- Activity modification - Analgesics/NSAIDs-Physiotherapy (flexibility and strengthening) - Custom orthoses - Temporary immobilization (cast/boot) [5,38]	Temporary pain relief and functional improvement [5]	Limited long-term efficacy, poor results once coalition ossifies [38]
**Coalition resection**	Failed conservative care; patients < 16 years; coalition < 50% of posterior facet; heel valgus < 16°; no subtalar arthritis [36,37,39,40]	- Open resection with interposition (fat, muscle, tendon, bone wax) [31,41,42,43,44] - Endoscopic/arthroscopic resection [45,46] - FHL tendon interposition [47,48]	- Significant pain reduction, improved AOFAS scores [46,49,50] - Return to sports common in adolescents [51,52,53] - High satisfaction; mean return to activity ~18 weeks [50]	Poor results if >50% facet involvement, valgus > 16°, degenerative changes [39,40]. Recurrence or progressive deformity is possible [53]
**Arthrodesis (subtalar/triple)**	Severe, extensive, or degenerative coalitions; adults; failed resection candidates [25,36,54,55,56]	- Subtalar arthrodesis - Triple arthrodesis	- Reliable pain relief and hindfoot stability [36,54,55] - Good functional outcomes, especially in adults [54,56]	Loss of subtalar motion Salvage option in severe deformities
**Emerging techniques**	Selected cases requiring minimally invasive or innovative approaches [45,46,57]	- Arthroscopic resection: smaller incisions, less soft-tissue disruption [45,46] - Percutaneous subtalar arthrodesis (PASTA) for medial facet coalition with normal alignment [57]	- Arthroscopic resection: 81% good/excellent outcomes, improved VAS and AOFAS, low complications [46] - PASTA: 100% fusion at 2-year follow-up, significant clinical improvement [57]	Evidence limited to mid-term results; need further studies [45,46,57]

## Data Availability

The data presented in this study are available on request from the corresponding author due to the inclusion of original data that are not publicly archived.

## Abbreviations

The following abbreviations are used in this manuscript:

AOFAS	American Orthopaedic Foot and Ankle Society
AP	Antero-posterior
CN	Calcaneonavicular
CT	Computed tomography
FDL	Flexor digitorum longus
FHL	Flexor hallucis longus
NSAIDs	Non-steroidal anti-inflammatory drugs
MRI	Magnetic resonance imaging
PASTA	Percutaneous subtalar arthrodesis
TC	Talocalcaneal
VAS	Visual Analogue Scale
